# Airborne geographical dispersal of Q fever from livestock holdings to human communities: a systematic review and critical appraisal of evidence

**DOI:** 10.1186/s12879-018-3135-4

**Published:** 2018-05-15

**Authors:** Nicholas J. Clark, Ricardo J. Soares Magalhães

**Affiliations:** 1 UQ Spatial Epidemiology Laboratory, School of Veterinary Science, The University of Queensland, Gatton, QLD 4343 Australia; 2 Children’s Health and Environment Program, Child Health Research Centre, The University of Queensland, South Brisbane, QLD 4101 Australia

**Keywords:** Airborne dispersal, *Coxiella burnetii*, Geographical contamination gradient, Spatial epidemiology, Q fever, Zoonotic disease

## Abstract

**Background:**

Q fever is a zoonotic disease caused by *Coxiella burnetii*. This bacterium survives harsh conditions and attaches to dust, suggesting environmental dispersal is a risk factor for outbreaks. Spatial epidemiology studies collating evidence on Q fever geographical contamination gradients are needed, as human cases without occupational exposure are increasing worldwide.

**Methods:**

We used a systematic literature search to assess the role of distance from ruminant holdings as a risk factor for human Q fever outbreaks. We also collated evidence for other putative drivers of *C. burnetii* geographical dispersal.

**Results:**

In all documented outbreaks, infective sheep or goats, not cattle, was the likely source. Evidence suggests a prominent role of airborne dispersal; *Coxiella burnetii* travels up to 18 km on gale force winds. In rural areas, highest infection risk occurs within 5 km of sources. Urban outbreaks generally occur over smaller distances, though evidence on attack rate gradients is limited. Wind speed / direction, spreading of animal products, and stocking density may all contribute to *C. burnetii* environmental gradients.

**Conclusions:**

Q fever environmental gradients depend on urbanization level, ruminant species, stocking density and wind speed. While more research is needed, evidence suggests that residential exclusion zones around holdings may be inadequate to contain this zoonotic disease, and should be species-specific.

**Electronic supplementary material:**

The online version of this article (10.1186/s12879-018-3135-4) contains supplementary material, which is available to authorized users.

## Background

The febrile illness “query fever” (Q fever; caused by the bacterium *Coxiella burnetii*) is a globally important zoonotic infection [1, 2]. This bacterium produces a unique cell (small cell variant, SCV) of high robustness, persistence and infectivity that resembles a bacterial spore [3]. Under experimental conditions, inhalation of a single SCV can produce infection, making *C. burnetti* one of the most contagious infectious agents known [3]. Human acute infections can be debilitating, commonly presenting with high fevers, pneumonia and / or hepatitis [4, 5]. While human fatalities are rare (fatality rates among untreated cases are around 1% [6]), outbreaks cause widespread health problems including endocarditis and associated heart failure, vascular aneurysms and chronic fatigue syndrome [7–9]. Importantly, up to 60% of chronic human infections caused by *Coxiella burnetii* are thought to be asymptomatic [10].

**Fig. 1:**
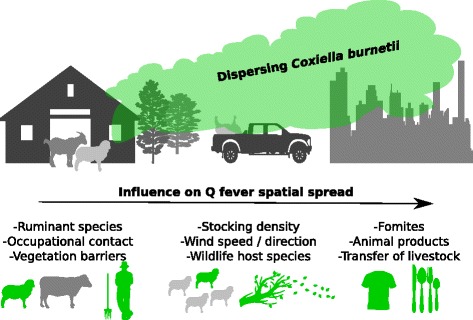
Schematic representation of potential drivers of *Coxiella burnetii* spatial dispersal from livestock holdings. Green shading indicates potential human transmission pathways. The top section of the figure demonstrates how airborne dispersal and environmental contamination are proposed to contribute to the zoonotic exposure of human communities. This dispersal can be influenced over a range of spatial distances by factors represented in the bottom section of the figure

Infected livestock are the primary sources of zoonotic Q fever outbreaks, though certain species have more prominent epidemiological roles. *Coxiella burnetii* can be recovered from goats and sheep (which are often asymptomatic) in large quantities in faeces, milk and vaginal mucus, amniotic fluid and other products of conception [11, 12]. Cattle rarely excrete by multiple routes [13]. Despite a widely available vaccine delivered to individuals with occupational risk (veterinarians, veterinary students and ruminant market workers on farms and in abattoirs [14, 15]), prominent Q fever notifications in absence of occupational exposure, together with evidence of exposure in non-livestock species and evidence that *C. burnetti* attaches to dust particles, suggests the existence of unmeasured transmission pathways [16–20] (see Fig. 1 for a schematic overview of potential transmission pathways). Environmental contamination likely plays a role, as several lines of evidence suggest the bacterium is more environmentally ubiquitous than once thought [17, 18]. *Coxiella burnetii* survives up to 10 months at 15 - 20 °C, > 1 month on meat in cold storage and > 40 months in skim milk at room temperature [21]. Moreover, *C. burnetii* can attach to dust particles, suggesting a prominent role of wind-borne dispersal [22]. Indeed, maintaining specific distances from animal holdings for housing developments is a common measure aimed at reducing geographical spread of Q fever [23, 24]. Yet while wind-borne dispersal is a probable Q fever risk factor [25] (Fig. 1), evidence for the geographical dispersal capability of *C. burnetii* is lacking.

The aims of this study were twofold: first, we assessed the strength of evidence for the role of distance from infected livestock facilities in human Q fever community outbreaks around the globe using a systematic search; second, we examine available evidence to evaluate the epidemiological factors and on-farm biosecurity measures that influence the likelihood of exposure of human communities to *C. burnetii*. We conclude by presenting informed recommendations for limiting Q fever outbreaks.

## Methods

Our central goal was to gather empirical evidence on the geographical dispersal potential of *C. burnetii*. We proceeded by searching the PubMed (coverage = 1966 to present), Medline (1966 to present), Web of Science (1900 to present) and Scopus (1970 to present) databases using combinations of keywords ‘Q Fever’, ‘Q-Fever’, ‘*Coxiella burnetii*’, ‘*C. burnetii*’, ‘distance’, ‘airborn*’, ‘aerosol’, ‘spatial’, ‘wind*’, ‘dispers*’, ‘gradient’, ‘human infection’, ‘outbreak’, ‘epidemic’, and ‘clinical presentation’ (see Additional files 1 and 2 for further details of literature search methods). We manually searched titles and abstracts to exclude papers that did not address human Q fever outbreaks or did not empirically analyse Q fever risk factors. Date of last search was 26th January, 2018.

From remaining papers, we searched full texts to identify studies that presented evidence on geographical gradients of Q fever infection. We adopted the PICO convention (Population, Intervention / Exposure, Comparison, Outcome) using the following inclusion criteria: Population = human communities; Exposure = human communities living and/or working within vicinities of potential source ruminant farms or abattoirs; Comparison = geographical distance to potential source ruminant farms or abattoirs; Outcome = documented human infections with Q fever.

For studies matching inclusion criteria, we extracted specific information to evaluate evidence for the role of distance from putative sources in human Q fever outbreaks. This included: livestock species housed at source, source type (abattoir or farm), livestock density at source, effect size of geographic distance on infection probability (if included), urban density (urban or rural) and information on other putative drivers of environmental dispersal (predominantly wind speed).

Due to low sample sizes, a general lack of reported effect sizes and the variety of analyses conducted by the included studies (see Results), we were not able to conduct a meta-analysis of the role of geographical distance in community human Q fever infection. We instead present a critical appraisal of evidence and make informed recommendations for future research and management practices.

## Results

### Evidence for Q fever geographical contamination gradients

A total of 298 papers were initially retrieved in our systematic search (full search results available in Additional file 3), and 18 of these (Table 1) presented empirical data on the geographical gradient of Q fever infection risk from putative farm sources (as of 26th January 2018). A further three papers presented data on geographical gradients from putative abattoir sources (Table 2). All included studies identified sheep or goat holdings as putative infection sources (i.e. no studies suggested cattle holdings were the primary infection source).

**Table 1:** Studies reporting estimated geographical dispersal potential from Q fever infected farms

Reference	Country	Year of outbreak	Urban density	Farm type	Farm size	Infective distance from animal holdings
[40]	Switzerland	1983	Rural	Sheep flocks	850-900 sheep	1 - 2 km
[32]^a^	Germany	2005	Urban	Gestating ewes	30 ewes	< 500 m; 60 m 14.7% +
[31]^a^	Germany	2005	Urban	Sheep farm	500 ewes; 35 lambing	11.8% attack rate within 50 m; 1.3% in the area 350 - 400 m
[29]^a^	Netherlands	2006-10	Urban (contaminated land parcels)	Goat manure	N/A	0 - 2.5 km: 52% +; 2.5 - 5 km: 30% +
[44]	France	2007	Rural	Sheep and goats	N/A	5 km
[61]	Netherlands	2007-9	Urban	Dairy goat farms	432-2653 goats	2 km
[24]^a^	Netherlands	2007-10	Urban	Dairy goat farms	> 50 goats	Most risk 0.5 - 1 km; acceptable risk of 50 cases per 100,000 for < 3 km
[30]^a^	Netherlands	2007-10	Urban	Dairy goat farms	N/A	Most risk < 4.1 km
[17]	Netherlands	2007-11	Urban	Multiple species regression	N/A	5 km
[25]	Netherlands	2008	Urban	Dairy goat farm	> 400 goats	2 km
[26]^a^	Netherlands	2009	Rural / Urban	Dairy goat farm	791 goats	Most risk < 5 km
[62]	Netherlands	2009	Urban	Three dairy goat farms	791 - 1295 goats	0.3 - 1.5 km
[63]	Netherlands	2009	Urban	Goats	2251-20,960 goats	5 km
[43]	Netherlands	2009	Urban	Dairy goat farm	450 pregnant goats	> 5 km
[64]	Netherlands	2009	Urban	Dairy goat farms and meat sheep farms	N/A	> 5 km
[27]^a^	Netherlands	2009	Urban	Dairy goat farms	N/A	1 km: 71% +; 5 km: 18% +; 10 km: 3% +
[28]^a^	Netherlands	2011	Urban (air samples 1-year post outbreak)	Goat farms	N/A	91 m: 56% +; 591 m: 25% +
[42]	Hungary	2013	Rural	Merino sheep flock	450 ewes	> 10 km

^a^indicates studies reporting specific effect sizes of geographic distance on the probability of infection

*N/A* information not available

**Table 2:** Studies reporting estimated geographical dispersal potential from Q fever infected abattoirs

Reference	Country	Year of outbreak	Urban density	Infective distance from animal holdings
[33, 36]	France (Briancon)	1996	Urban	Increased risk at 250 m compared to 1 km distance of exposure
[35]	France (Marseilles)	1999-2002	Urban	< 2 km; Wind speeds 28-36 km/h noted

Estimated distances of Q fever contamination from putative farm sources ranged from < 1 km to > 10 km (Table 1). Studies in rural areas (*N* = 4) indicated the highest infection risk generally occurs within distances of 5 - 10 km of infected farms; most urban outbreaks generally identified smaller distances, with highest risk occurring within 2 - 4 km (Table 1). For instance, when assessing the urban outbreak in The Netherlands, living < 2 km from a dairy goat farm where a *C. burnetii* abortion wave had occurred was identified as the most important risk factor of Q fever infection probability [25]. Specifically, persons living within 2 km of a dairy goat farm (defined as a property with > 400 animals) with abortion problems posed a higher risk for Q-fever than those living more than 5 km away (Relative risk: 31.1 [95% confidence interval: 16.4 - 59.1]). Another study investigating the same outbreak used data from several sources and sectors to investigate 17 farms in the area as probable sources using GIS mapping and smooth incidence of cases [26]. Their analyses indicated that persons living within 1 km of the putative source farm were at a 46 times larger risk of being a case compared to those living within 5-10 km. Despite these prominent examples, the specific influence (i.e. effect size) of geographical distance from putative farm sources was reported in only eight studies (Table 1). Six of these assessed putative goat farm sources during the outbreak in The Netherlands and were broadly in agreement, finding that persons living within 1 km of a source were at larger infection risk compared to those living within 5 - 10 km [24, 26–30]. A study by Commandeur et al. [26] provides an overview of policy decision challenges around distancing of human communities from goat farms, proposing that an acceptable incidence rate of 50 human cases per 100,000 people occurs > 3 km from sources. In contrast, de Rooij et al. [31] found a steeper gradient in infection risk for the urban outbreak in The Netherlands, with persons living / working 591 m from the source at half the risk compared to those living / working within 90 m.

The two remaining studies reporting effect sizes for geographical distance from farms were from an urban outbreak in Jena, Germany in 2005 [31, 32], where 331 human cases were reported from a residential area and were associated with proximity to a meadow where sheep were grazing and lambing. Both studies found lower distances of Q fever geographical dispersal compared to the outbreak in The Netherlands, with infection probabilities rapidly dropping off at distances greater than 500 m from the putative source (Table 1).

The three studies reporting distance to abattoirs as a risk factor came from France; one from Marseilles and two from the region of Briancon (Table 2). None of these reported effect sizes, though the study by Carrieri et al. [33] did include distance as part of a metric to describe level of exposure, with findings indicating that increased exposure (linked to closer distances to the source, in addition to other exposure variables) led to increased risk of infection.

### Wind as an important epidemiological factor

Wind has been implicated as an epidemiological factor in the spread of Q fever in studies from farm and abattoir putative sources (Fig. 1; Tables 2 and 3). A study using an atmospheric dispersion model for the outbreak in The Netherlands demonstrated that wind speeds exceeding threshold values of 2 m/s in the vicinity of goat farms were associated with outbreaks [34]. A study from Marseilles, France used wind direction to identify an abattoir used to slaughter sheep for the Eid al-Adha festival (also known as Festival of Sacrifice). as the putative source [35]. This abattoir was located 2 km downwind from the affected homeless shelter. Dry and windy weather conditions are suggested to have facilitated the spread of outbreaks occurring in Bulgaria, France and Germany [10]. In addition to natural wind, two studies of an outbreak in Briancon, France indicate that airborne transmission of *C. burnetii* from contaminated goat and sheep waste, left uncovered in the slaughter area, was likely driven by aerosolised particles due to operation of helicopters from a nearby heliport [33, 36]. In addition, aerosolisation of *C. burnetii* through manure spreading has also been implicated as a risk factor for human outbreaks [29].

**Table 3:** Studies reporting the role of wind in the spatial dissemination of *Coxiella burnetii* from Q fever infected farms

Reference	Country	Year of outbreak	Wind Speed	Distance
[45]	France	1998	> 8 m/s	Radius of 20 km
[31, 32]	Germany	2005	11-18 m/s	< 500 m
[25]	Netherlands	2007	–	< 5 km; mostly < 2 km
[46]	UK	1989	130 km/h	< 18 km

### Other documented environmental factors influencing Q fever transmission

Most community outbreaks have been associated with the lambing / calving period of goats or sheep and have been temporally linked to the lambing season in high density rearing areas [1, 37] (Fig. 1). Indeed, from a range of studies covering outbreaks in Hungary, Germany, Switzerland, France and The Netherlands, high-density sheep rearing was commonly considered a risk factor (Table 1). Note however that the way density is defined is not consistent across studies; generally, a threshold of > 400 goats is considered ‘high density’ (Table 1).

The movement of domestic ruminants and their products (either for consumption or via fomites) has been linked to propagated human outbreaks in Bulgaria, France, Switzerland, Britain and The Netherlands [38, 39] (Fig. 1). Two outbreaks in Britain and Switzerland demonstrated that residents living along roads through which vehicles of sheep travelled could become infected as a result of exposure to contaminated straw or dust [40].

## Discussion

Identifying the geographical dispersal potential of *C. burnetii* is important for developing a better understanding of Q fever outbreaks. Our review suggests that in rural areas, the highest risk of infection occurs within 5 km of infected farms, whereas urban outbreaks generally occur within smaller distances, with the highest risk in areas 2 - 4 km from source farms. Probable reasons for this inconsistency are factors that modulate geographical dispersion, including wind speed, timing of outbreak (e.g. synchronicity with goat / sheep outbreaks) and presence of landscape features such as vegetation barriers. More targeted research on geographical dispersal is clearly needed, particularly since Q fever epidemiology studies generally do not provide detailed information about infection risk as a function of geographical distance from the source. Nevertheless, our findings indicate that current exclusion zone recommendations may not be adequate to prevent Q fever outbreaks from livestock sources. On-farm control measures, particularly during lambing and calving periods for sheep and goats, will play a major role in limiting the spread of *C. burnetti*.

### Distance decay and the role of wind dispersal in human Q fever outbreaks

Our results demonstrate that while geographical distance from livestock sources is a potentially important risk factor in Q fever spatial epidemiology, is has been largely understudied. The best accounts of spatial Q fever risk decay are provided by studies of the Jena, Germany outbreak in 2005 [31, 32] and the 2009 outbreak in The Netherlands [24, 26, 27]. In Germany, the attack rate was 11.8% within 50 m, decreasing with growing distance to 1.3% at 350 - 400 m. This evidence, along with results from Commandeur et al. [24] implying dairy goat farming should not occur within 3 km of residential dwellings, forms the basis for recommendations by the Robert-Koch Institute to the German planning authorities for a 500 m residential construction exclusion zone around sheep rearing areas. Similar recommendations are in place in Queensland, Australia, where regulatory controls state “Town planning should consider the potential for windborne spread of Q fever and limit the encroachment of residential dwellings on existing likely sources of Q fever including abattoirs, tanneries, and stockyards. The recommended buffer zone between residential dwellings and these types of facilities is at least 1km.” [41]. This advice comes from Heymann [23], which suggests airborne *C. burnetii* particles can be carried downwind for > 1 km.

While several urban outbreak studies provide some support for 1 km exclusion zones, our results generally suggest this distance is inadequate, particularly for outbreaks linked to small ruminant farming (i.e. goats and sheep). Infections during rural and urban outbreaks are estimated to occur 5 or even 10 km from these sources [32, 42–44]. Such broad contamination zones support mounting evidence that wind is an important component of the *C. burnetti* dispersal kernel [22, 45]. Indeed, wind was noted in the earliest report of the role of distance on human Q fever infection using a geographic information system, which came from the United Kingdom [46]. This study described an outbreak of human Q fever in the West Midlands in 1989. This outbreak was due to unusual southerly gales of up to 125 km/h in combination with the occurrence of lambing events throughout the region. Our results expand on this to show that wind may also facilitate outbreaks from infective animal products. For example, a small outbreak in France was likely caused by aerosol transmission from goat and sheep manure infected with *C. burnetii* applied to nearby pastures [44]. This suggests that proximity should not be seen in isolation, as wind speed will interact with geography to influence the spread of infection [27, 47].

Limiting factors in recommending appropriate residential exclusion zones include the fact that most of the studies we identified did not report estimated effect sizes, nor where they designed to demonstrate geographical dispersal as a putative risk factor. This makes it difficult to draw generalisable conclusions about geographical gradients in attack rates. Another important confounder of the large estimated dispersal distances we uncovered is the role of fomites, such as clothing and other materials, which are potentially moved from infected areas. Movement of infectious *C. burnetii* particles, which are known to survive for long periods in the environment, across relatively large distances will make estimation of airborne dispersal kernels difficult. In addition, the studies included in this review used infection data based on notified cases (i.e. patients with clinical signs), which means that infections are likely to be more geographically widespread. Reported incidence will depend on the sensitivity and specificity of diagnostic tools used, as well as the interest of practicing clinicians and the awareness of the general public [10].

### Other risk factors for geographical dispersal of Q fever

Our results demonstrate that the risk of propagated human outbreaks is influenced by livestock species. We found no evidence to support a major contribution of cattle to propagated human Q fever outbreaks, despite existing evidence of *C. burnetti* shedding by infected cattle [13]. This finding supports previous evidence that propagated human outbreaks (outside occupational exposure) are commonly attributed to infective sheep and goats [48]. Although reasons behind this difference between sheep / goats and cattle as outbreak sources are not entirely clear, it may be due to: (1) the highly seasonal nature of their reproduction cycles; (2) their comparatively large herd sizes; (3) differences in husbandry/biosecurity systems; (4) the relative importance of shedding and abortions after *C. burnetii* infection. Infective sheep and goats can suffer abortion waves and shed *C. burnetii* in subsequent pregnancies [49], with goats being particularly susceptible [12]. In contrast, abortion waves in infected cattle are less common [50]. However, infected cattle may still pose a zoonotic risk through *C. burnetii* excretion in milk [11, 13].

In addition to the type of species housed at source facilities, a number of other factors can influence the geographical dispersal of Q fever. Environmental forces in addition to wind may indirectly contribute to outbreaks. For example, rainfall totals may contribute to dust production and have an influence on the timing of lambing / calving seasons [45] or the local abundances of wildlife reservoirs [51]. Movement of infected animals and/or their products may lead to Q fever transmission through two separate mechanisms – seeding of infected animals in different locations and b) aerosolisation of *C. burnetti* during transit. Indeed, studies have shown that manure from Q fever-positive dairy goat farms may contain high concentrations of *C. burnetii*, which could be an important source for aerosolisation [2, 12]*.* Other risk factors include the type of enterprise (abattoir vs farm), native wildlife that are exposed to infection and can act as effective carriers, and fomites transported through infectious clothing or farm gear [18, 52]. Indeed, a broad range of wildlife species have been found to be susceptible to *C. burnetii* infection (including many mammalian species and blood-feeding ticks; [18, 53, 54]). Yet to our knowledge, the role of non-domestic hosts in the geographical spread of Q fever has not been studied. Our understanding of *C. burnetii* transmission pathways (and indeed, of many multi-host pathogens that utilize wildlife reservoirs) remains incomplete, especially in regard to maintenance within non-ruminant species [10, 52, 55–58].

### Management plans for the mitigation of *C. burnetii* spread to urban communities

Q fever management plans within livestock facilities need to be multifaceted to mitigate the risk of propagated human outbreaks. However, the relative efficacy of on-farm Q fever control measures at limiting geographical dissemination of *C. burnetii* is not well documented [48]. We postulate that exposure of communities to *C. burnetti* occurs as a result of two processes: first, the release of *C. burnetii* to the environment and second, the existence of factors that allow communities to come into contact with contaminated environment. Contact with contaminated environment will primarily be a function of the adequacy of existing biosecurity controls at animal holdings. Given our evidence of potential environmental contamination by *C. burnetti* to areas outside a 1 km radius from a source, measures to reduce dispersion potential from livestock enterprises may help prevent human outbreaks. The dispersion of *C. burnetii* from infected animal holdings can be limited by enhanced farm bio-exclusion measures such as indoor parturition, safe disposal of parturition materials and in the case of abattoirs the safe disposal of hides and offal. Managing soil properties to reduce dust production such as increasing soil moisture through irrigation or concreting surfaces will be effective strategies to at reduce aerosolisation. Furthermore, the placement of high vegetation barriers around animal holdings has been suggested to reduce the risk of transmission of *C. burnetii* to human communities [47]. Controlling contact rates between farmed animals and surrounding wildlife, as well as preventing transport of potentially infectious material and personnel clothing, should be maintained.

The spread and severity of *C. burnetii* outbreaks in humans can also be reduced by within-farm medical and sanitary measures such as vaccination and improvement of on-farm hygiene practices. A recent study found that Australia’s Q fever vaccination program has reduced notification rates by more than 50% [16]. However, this study, together with multiple other studies, demonstrated that the program suffers from poor coverage in some at-risk groups, particularly among farmers and veterinary nurses [16, 59]. Available evidence on the efficacy of goat or dairy goat vaccination indicates that an inactivated phase I vaccine reduces risk of shedding from reproductive track secretions in previously sensitized goats compared [60]. Bontje et al. provide evidence to support this in a study that used a mathematical model to assess the relative efficacy of on-farm Q fever control strategies to reduce the cumulative amount of *C. burnetii* in dried dust emitted into the environment from goat farms [60]. They found that the most effective control strategy is preventive yearly livestock vaccination, followed by reactive strategies to vaccinate animals within the herd after an abortion wave or after positive bulk tank milk tests. This study also demonstrated that while culling of pregnant goats during an abortion wave reduces concentrations of *C. burnetii* emitted into the environment, emission is not entirely preventable and Q fever will not be eradicated. Finally, the authors reported that eradication of Q fever in goat herds that excrete *C. burnetii* intermittently will not be achieved by a test (e.g. PCR of individual milk samples) and cull strategy.

## Conclusions

Airborne dispersal kernels for Q fever have been estimated in the order of 5 km or even 10 km from putative sources. Based on this evidence, our study indicates that residential exclusion zones smaller than 2 km around livestock enterprises may not be adequate to prevent propagated Q fever human outbreaks. These estimates are heavily species-specific, as previous evidence shows that propagated human outbreaks (outside occupational exposure) are commonly attributed to sheep and goats, but not cattle. While studies demonstrate that dispersion of *C. burnetii* from infected farms can be limited by multifaceted management plans including enhanced bio-exclusion measures (including indoor parturition, safe disposal of parturition materials, hides and offal, and measures to reduce dust formation and aerosolisation), the relative efficacy of these measures is largely unknown.

## Additional files

Additional file 1: Extracting search results for possible distance-decay functions of Q fever recovery. A .pdf file presenting R code for performing PubMed searches (using functions in Additional file 2) for possible distance-decay functions of Q fever and collating equivalent search results from additional databases into a single dataframe. (PDF 135 kb)

Additional file 2: R functions to perform PubMed searches and collate results. A zipped file containing R functions to perform PubMed literature searches and collate equivalent search results from additional databases into a single dataframe. (ZIP 4 kb)

Additional file 3: Raw literature search results. A .csv file containing data on the 298 research articles identified in primary searches. (CSV 533 kb)
