# Supplementary material for: Airborne geographical dispersal of Q fever from livestock holdings to human communities: a systematic review and critical appraisal of evidence
Source: BMC Infect Dis. 2018 May 15;18:218. doi: 10.1186/s12879-018-3135-4 (PMC5952368; doi:10.1186/s12879-018-3135-4)
Supplement: Supplementary file 1 — Extracting search results for possible distance-decay functions of Q fever recovery. A .pdf file presenting R code for performing PubMed searches (using functions in Additional file 2) for possible distance-decay functions of Q fever and collating equivalent search results from additional databases into a single dataframe. (PDF 135 kb) [file 12879_2018_3135_MOESM1_ESM.pdf]

Extracting search results for possible distance-decay functions of Q fever recovery

Nicholas J Clark (nicholas.j.clark1214@gmail.com), Ricardo Soares Magalhães (r.magalhaes@uq.edu.au)

This appendix replicates methods used gather references from a list of search terms, primarily with functions that utilise the 'rentrez' R package

Load searching functions in the PubMed_extractions.R script

```
source("PubMed_extractions.R")
```

Create list of search terms using PubMed syntax

```
term.list = c("(Q Fever OR Q-Fever OR coxiella burnetii OR c. burnetii)
              AND (distance OR airborn* OR aerosol) AND (human infection
              OR outbreak OR epidemic OR clinical presentation)",
    "(Q Fever OR Q-Fever OR coxiella burnetii OR c. burnetii)
              AND (spatial OR wind* OR dispers* OR gradient) AND
              (human infection OR outbreak OR epidemic OR clinical
              presentation)")
```

Search PubMed for all the search terms and return a dataframe of unique (i.e. unduplicated) records

```
pmed.search <- PubMed_extractions(term.list = term.list,
    retmax = 750)
```

Table 1: Preview of PubMed search results

title	authors	year	journal
A RAPID BIO-OPTICAL SENS...	Koo, Bonhan, Ji...	2017	JOURNAL OF BIOPHOTONICS
ATYPICAL OUTBREAK OF Q F...	Archer, Brett N...	2017	COMMUNICABLE DISEASES IN...
COXIELLA BURNETII IMMUNO...	Gerlach, C, Šku...		ACTA VIROLOGICA
PROTEIN COMPOSITION OF T...	Flores-Ramírez,...		ACTA VIROLOGICA
TRANSMISSION OF COXIELLA...	Bechah, Yassina...	2017	COMPARATIVE IMMUNOLOGY, ...
CLIMATE CHANGE EFFECTS O...	van Leuken, J P...	2016	AEROBIOLOGIA
VASODILATOR-STIMULATED P...	Colonne, Punsir...	2016	PLOS PATHOGENS
MURINE ALVEOLAR MACROPHA...	Fernandes, Tali...	2016	INFECTION AND IMMUNITY
MOUSE MODEL OF COXIELLA ...	Melenotte, Cléa...	2016	INFECTION AND IMMUNITY
SPREAD OF COXIELLA BURNE...	Pandit, Pranav,...	2016	VETERINARY RESEARCH
HUMAN Q FEVER INCIDENCE ...	Van Leuken, J P...	2016	ONE HEALTH (AMSTERDAM, N...
DETECTION OF COXIELLA BU...	de Rooij, Myrna...	2016	PLOS ONE
DEVELOPMENT OF AN EX VIV...	Graham, Joseph ...	2016	INFECTION AND IMMUNITY
Q FEVER	Shishido, Akira...		U.S. ARMY MEDICAL DEPART...

Read in the tab-delimited results from equivalent searches in Web of Science (i.e. using identical Boolean search chains). In this case, these search results are stored as multiple .txt files, all including the phrase "Distance_decay_wossearch" in the file name

```
source("Read_engine.searches.R")
WOS.df = Read_WOS.searches(path = "./Lit search results raw/",
    pattern = "Distance_decay_wossearch")
```

Read in the comma-separated results from equivalent searches in Scopus and Medline (i.e. using identical Boolean search chains). In this case, these search results are stored as multiple .csv files, all including key phrases in the file names

```
Scopus.df = Read_Scopus.searches(path = "./Lit search results raw/",
    pattern = "Distance_decay_scopussearch")
Medline.df = Read_Medline.searches(path = "./Lit search results raw/",
    pattern = "Distance_decay_medlinesearch")
```

Bind all of the search result dataframes together and remove duplicates

```
all.searches <- Bind_searches(list(WOS.df,
    pmed.search, Scopus.df, Medline.df))
```

How many unique papers have we found in total?

```
nrow(all.searches)
```

```
## [1] 217
```

Write the results as a .csv file for visual processing of abstracts and titles

```
write.csv(all.searches, file = paste("/Distance_decay_pmedwossearch_",
    format(Sys.time(), "%Y-%m-%d"), ".csv",
    sep = ""), row.names = T)
```
